# The impact of state cannabis legislation, county-level socioeconomic and dog-level characteristics on reported cannabis poisonings of companion dogs in the USA (2009–2014)

**DOI:** 10.1371/journal.pone.0250323

**Published:** 2021-04-16

**Authors:** Mohammad Howard-Azzeh, David L. Pearl, Alexandra Swirski, Madeline Ward, Roksolana Hovdey, Terri L. O’Sullivan, Olaf Berke

**Affiliations:** 1 Department of Population Medicine, University of Guelph, Guelph, Ontario, Canada; 2 Department of Mathematics and Statistics, University of Guelph, Guelph, Ontario, Canada; University of Lincoln, UNITED KINGDOM

## Abstract

With current trends in cannabis legalization, large efforts are being made to understand the effects of less restricted legislation on human consumption, health, and abuse of these products. Little is known about the effects of cannabis legalization and increased cannabis use on vulnerable populations, such as dogs. The objective of this study was to examine the effects of different state-level cannabis legislation, county-level socioeconomic factors, and dog-level characteristics on dog cannabis poisoning reports to an animal poison control center (APCC). Data were obtained concerning reports of dog poisoning events, county characteristics, and state cannabis legislation from the American Society for the Prevention of Cruelty to Animals’ (ASPCA) APCC, the US Census Bureau, and various public policy-oriented and government websites, respectively. A multilevel logistic regression model with random intercepts for county and state was fitted to investigate the associations between the odds of a call to the APCC being related to a dog being poisoned by a cannabis product and the following types of variables: dog characteristics, county-level socioeconomic characteristics, and the type of state-level cannabis legislation. There were significantly higher odds of a call being related to cannabis in states with lower penalties for cannabis use and possession. The odds of these calls were higher in counties with higher income variability, higher percentage of urban population, and among smaller, male, and intact dogs. These calls increased throughout the study period (2009–2014). Reporting of cannabis poisonings were more likely to come from veterinarians than dog owners. Reported dog poisonings due to cannabis appear to be influenced by dog-level and community-level factors. This study may increase awareness to the public, public health, and veterinary communities of the effects of recreational drug use on dog populations. This study highlights the need to educate dog owners about safeguarding cannabis products from vulnerable populations.

## Introduction

The lethality of cannabis on dogs is poorly understood [1] and there is no established minimum lethal dose (MLD) or median lethal dose (LD_50_) [2]. Additionally, co-ingredients in products containing cannabis, such as chocolate, confound the clinical picture [3]. A growing body of evidence is supporting both the potential for substantial adverse effects in dogs from the consumption of different cannabis associated chemicals, namely tetrahydrocannabinol (THC) [2–5], as well as those involved in the therapeutic use of different cannabis products, such as cannabidiol (CBD) [6–13]. With several countries and subnational states recently legalizing cannabis possession and use, and with many other governing bodies claiming to soon follow suit, it is prudent to have a clearer picture of the relationship between cannabis and accidental poisoning in vulnerable populations who’s health and safety relies on that of their caretakers, like companion animals.

Several studies have examined the impact of legalized cannabis possession on human populations [14–17], although more research is needed [18]. There are concerns that legalization may increase substance abuse [17, 19], and lead to an increase in unintentional poisoning events in other vulnerable populations such as children [20]. The veterinary community needs to be prepared for similar issues involving dog populations [21]. However, the effects of legalized cannabis possession on dogs, has received little attention. Previous studies have noted a positive association between dog owners’ medical cannabis licenses and cannabis toxicosis in dogs [21], as well as an increase in accidental exposure to cannabis products in dogs [3]. These reports support the concern that legislation may impact the risk of cannabis poisoning events in dogs. To date, no study has examined the impact of cannabis legislation on dogs specifically.

There is a large body of research showing an association between socioeconomic factors and cannabis use and use disorders [22–26]. Increased cannabis use is associated with several socioeconomic variables, such as lower-income, lower financial stability, lower education, lower relationship and life satisfaction as well as higher welfare dependence [23, 26]. We suspect an association between a dog’s risk of cannabis exposure and its socioeconomic environment.

Dog characteristics such as breed, sex, size, and reproductive status are associated with accidental dog poisoning events from other toxicants, such as insecticides and opioids [27, 28]. It is likely the same characteristics are associated with cannabis exposures in dogs. Therefore, the objectives of this study were to identify the impact of cannabis legislation, human socioeconomic variables, and dog-level characteristics on the odds of a cannabis poisoning related call to an animal poison control center in the United States (US).

## Methods

### Data

Dog-level variables used in this study were collected by the American Society for the Prevention of Cruelty to Animals (ASPCA). The ASPCA operates the Animal Poison Control Center (APCC) that provides over-the-phone emergency toxicology advice to those needing assistance administering care to a potentially poisoned animal. The services provided by the APCC cost 65 USD and may be used by the public, veterinarians, or other poison control centers. The APCC collects data from each call concerning the number of animals exposed, patient signalment, clinical effects, outcome, toxicant information, and date/location/time of the call. The data are stored in the APCC’s "AnTox" toxicology database. This study used only AnTox data from January 1^st^, 2009 until December 31^st^, 2014.

Each call to the APCC regarding a dog patient was considered a unique observation. A case was defined as any call to the APCC that involved a dog exposed to cannabis or a cannabis derivative. These included all forms of cannabis such as: raw cannabis regardless of species (n = 1315), synthetic cannabinoids (n = 68), THC (n = 97), CBD (n = 2), hash oil (n = 4), and hemp seed oil (n = 1). These products were often mixed with other products, such as edible chocolate brownies. It was also considered a case, if a dog was exposed to cannabis and another toxicant at the time the call was reported. A control was a call to the APCC involving a dog that was exposed to any non-cannabis toxicant. Each case or control represents a single dog. Route of exposure was not considered in this study. Once a poisoning event is logged by the APCC, then any following correspondence (i.e., with veterinarians or owners) is linked to the event.

The data used in the study from the AnTox database included 133,309 unique events. The dog-level variables of interest, extracted from the AnTox database were: age (years), weight (kg), reproductive status, sex, breed, toxicant exposure, year, call source, and the latitude/longitude of each caller to identify the county and state of the call’s origin.

The AnTox database contained information on each dog’s primary/apparent breed. This information was used to assign dogs into the following American Kennel Club (AKC) breed classes: herding, hound, Foundation Stock Service (FSS), non-sporting, sporting, terrier, toy, working, and other. Dogs that fell under the AKC’s miscellaneous category (n = 24) were classified as part of the FSS category. The AnTox database contained a field for describing each dog’s breed as mixed, pure, or if the owners were not asked. Approximately 72% of the observations had the field marked as “not asked”, therefore the purity of the breed was not considered and only the primary/apparent breed was used to classify the breed class of each dog.

Observations for the age and weight variables were treated as missing data if an implausible value was recorded. Ages recorded as “0” (n = 831) or greater than 26 years old (n = 9) were not used in this study. Weights recorded as “0” (n = 812) or exceeding 114 kg for giant breed dogs (Great Danes, Mastiffs, Neapolitan Mastiffs, Tibetan Mastiffs, Leonbergers, Boerboels, Newfoundlands, St. Bernards) (n = 0) or exceeding 75 kg for all other breeds were not used in this study (n = 17).

The original coding in the AnTox database for the reproductive status variable was immature, neutered, intact, pregnant, lactating, or unknown. This coding was used to determine the categories used in the analysis: intact, neutered, or unknown. The sex variable was originally coded in the AnTox database as female, male, did not ask, group, and unknown. These data were used to determine if the dog was female, male, or unknown, the categories used to classify sex in this study.

The source of the call to the APCC was recorded in the AnTox database as public, veterinarian, other poison control center (n = 7), and Animal Product Safety Service (n = 36). Only calls from veterinarians and the public were used in this study.

The following county-level socioeconomic variables were collected from the American Community Survey: percent bachelor’s degree or higher (age 25 and older), percent high school diploma or higher (age 25 and older), percent unemployed, percent did not work (in the last year), percent divorced, percent married family households, median age, median housing cost to income ratio, age dependency ratio (ratio of individuals aged 0–14 and over 65 compared to the total population), and sex ratio (number of males per 100 females). In the U.S., counties are sub-state administrative areas that are made up of a group of cities and/or towns and their surrounding areas.

Data concerning these variables were available for the entire study timeline. County-level Gini index information was also taken from the American Community Survey, but data were only available from 2010–2014. Therefore, 2009 Gini information was substituted using Gini data from 2010 for each county. A Gini index of 1 shows absolute wealth inequality, where all the wealth in a community is owned by a single person. A Gini index of 0 shows absolute wealth equality, where everyone has the same wealth. These values were used to create the income disparity variable used in this study.

The 2010 US Census was used to obtain information about a county’s percent urban population as well as racial distribution. Census racial distribution information (Hispanic, White, Black, American Indigenous, Asian, Native Hawaiian and other Pacific Islander, Other, two or more races) was used to calculate a Shannon diversity index to capture each county’s ethnic diversity. Shannon diversity index takes into consideration the proportion of ethnic groups as they are defined. If all of the individuals in a community are mostly made up of one ethnic group and the other ethnic groups are very rare, the Shannon index approaches 0. Since the ethnic diversity variable is calculated using 8 different ethnic groups, if all ethnic groups in a county were in equal proportions, the maximum Shannon index value would be 2.08. Data for urban and diversity variables were only available for 2010, therefore these data were used to characterize their respective counties for all years of the study.

Data concerning state-level legislation on recreational and medical cannabis use and possession were obtained from websites such as the National Organization for the Reform of Marijuana Laws (norml.org) and Marijuana Policy Project (mpp.org) from 2009–2014. Policy briefings from the West Virginia Center on Budget & Policy, archived state codes, State-by-State Medical Marijuana Laws Report (Marijuana Policy Project), and public bills from state legislative websites were also used to identify state legislation changes at given time points. As state legislation changed throughout the period of this study, changes in legislation were noted in the year they were enacted, regardless of the month when the change occurred. Typically, state policy regarding cannabis use is divided into felony, misdemeanour, decriminalized, and legal as defined by fine amount and prison length. Felony and misdemeanour charges carry potential fines, recording of a criminal record, and prison sentences of varying length. Furthermore, some states allow medical cannabis exceptions, regardless of the severity of the penalty for non-medical possession. Consequently, we categorized the legislation variable into three groups: “restricted” if cannabis possession was a felony or misdemeanour, “restricted plus medical” if cannabis possession was a felony/misdemeanour but medical use was permitted, and “legalized” if cannabis possession was legal or decriminalized (regardless of medical legality). The penalty for the lowest infraction was used to classify states with multiple levels of penalty, usually for possession under a small amount ranging from 14.17 g to 113.40 g, and first offence. Additionally, different US governing bodies have banned specific synthetic cannabinoids and general categories of chemicals in an attempt to control the use of dangerous synthetic cannabinoids. Makers of synthetic cannabinoids try to evade these laws by creating new synthetic cannabinoid molecules that are not yet regulated. The legal status variable only includes legislation controlling naturally occurring forms of cannabis, and does not include synthetic cannabinoids.

### Statistical analysis

Descriptive statistics including means, medians, interquartile ranges, standard deviations, and 95% confidence intervals were performed. However, all descriptive statistics were reported based on the type of data (i.e., nominal, ordinal, or continuous) used for subsequent multi-level modelling. The correlation between independent variables was examined using correlation coefficients (i.e., Pearson, Phi, and Spearman’s rank) depending on the type of independent variables. If the correlation between two variables was greater than |0.75|, the more epidemiologically plausible variable was kept in the model moving forward. Linearity between continuous independent variables and the log odds of being a cannabis-related call was assessed graphically using locally weighted scatterplot smoothing (LOWESS) curves. If the relationship was not linear, the independent variable was categorized, or if appropriate, was modeled as a quadratic relationship with the addition of a squared term. Univariable mixed logistic regression models between the independent variables and the log odds of a call concerning a dog cannabis poisoning event were fitted to evaluate their associations. Independent variables with significant associations (α = 0.05) were considered for inclusion in a multivariable model. The dataset (n = 133,266) was analyzed using Stata 15 (StataCorp, College Station, TX).

Throughout the statistical modelling process, hierarchical random intercepts for county and state were added in all univariable and multivariable models to account for clustering. There were more than 10,000 calls reporting dog poisonings that involved more than one dog. Adding a hierarchical random intercept for each household to control for clustering at the household-level caused problems with model convergence. Therefore, one randomly chosen animal was included in the analysis from calls involving a household with several dog poisonings.

**Fig 1:**
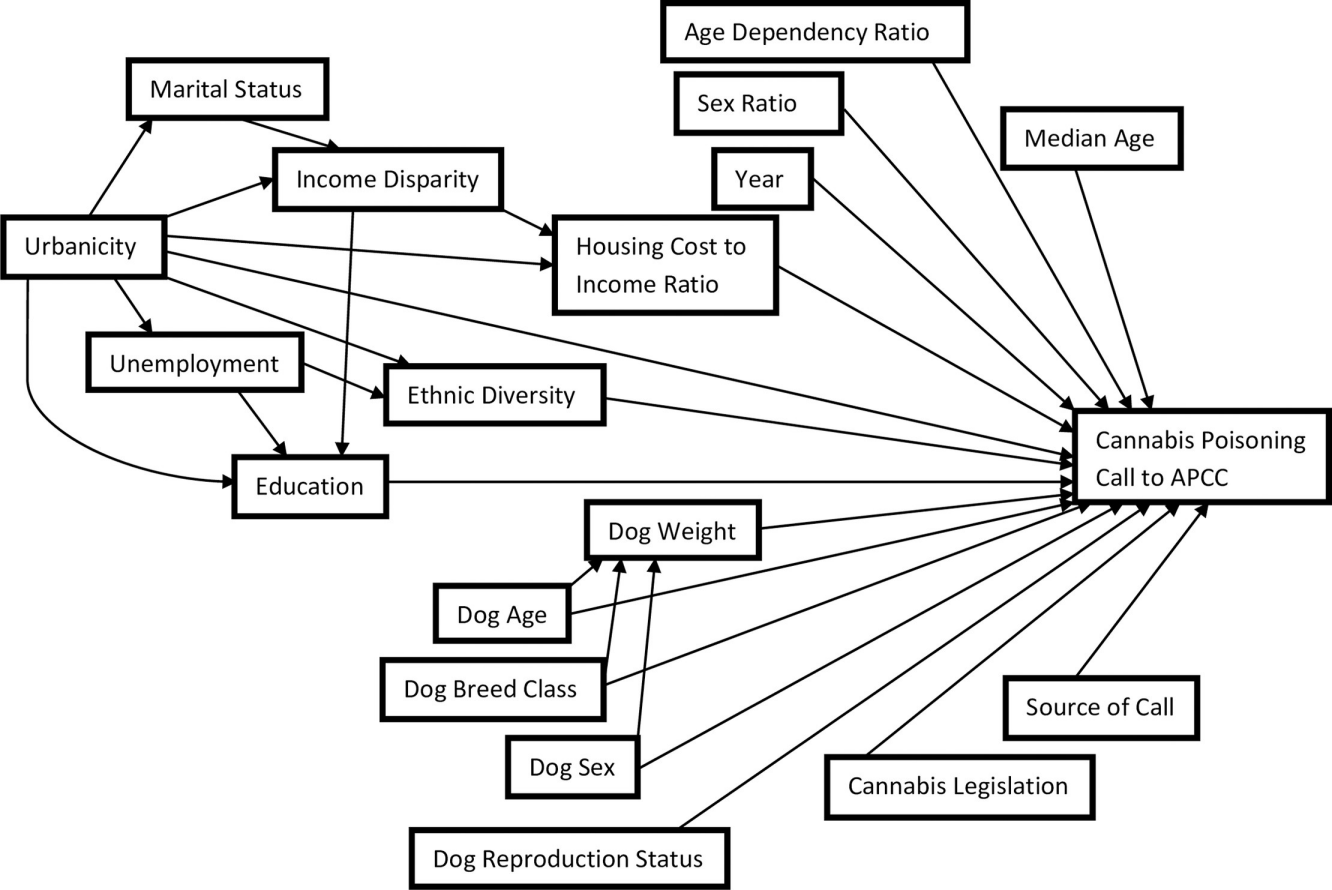
Causal diagram representing the relationship between dog-level and community-level factors and the odds of a call being related to a dog poisoning event from a cannabis product.

Forward variable selection was applied in mixed logistic regression modeling. Predictor variables were added to the model one at a time from most to least significant based on univariable analyses. Biologically plausible two-way interactions at the dog-level (weight, breed class, sex, and reproductive status) and county-level (percent urban population, ethnic diversity, income disparity) that were identified *a priori* (Fig 1) were assessed one at a time in the main effects model. Variables with more than two categories had their overall significance tested with a Wald’s χ^2^ test. Variables that did not meet the statistical criteria in the forward model building process were re-introduced to the model, and if a given variable caused a 20% change or greater in any coefficient of another significant variable on its re-introduction, it was considered an explanatory antecedent (i.e., confounder if effect reduced) or distorter variable (i.e., effect increased or direction of association changed), given it met the causal criteria (i.e., non-intervening variable) based on the causal diagram (Fig 1). Predictor variables were included in the final model if they were statistically significant (α = 0.05), were part of a statistically significant interaction, or acted as an explanatory antecedent or distorter to another predictor variable. Outliers were assessed using Pearson and deviance residuals. The normality and homoscedasticity assumption for the random intercepts were assessed using their respective best linear unbiased predicted values at the state and county levels. Variance partition coefficients at the dog, county, and state-levels were estimated from the variance components from the final model using the latent variable technique [29].

Due to concerns over the misuse of the term “statistically significant” [30], in this manuscript, the term “statistically significant” does not suggest causation or epidemiological/biological importance. It is used to indicate that based on our statistical criteria, we have enough evidence to infer that the measure of association for a given predictor variable or contrast is different from the null value [28]. We consider the term “statistically significant” in an exploratory rather than confirmatory sense [31].

## Results

### Descriptive statistics

Of all dog calls to the APCC, 1.12% (n = 1,487) were related to cannabis. From 2009 to 2014, annual cannabis calls to the APCC increased from 0.84% (n = 179) to 1.53% (n = 362) of all toxicant calls (Table 1). The proportion of cannabis calls increased from 2009 to 2011. The proportion of cannabis calls decreased in 2012, then increased until the end of the study in 2014 where it peaked.

**Table 1:** Descriptive statistics concerning the frequency of calls made on behalf of US dogs to the APCC^a^ for cannabis and non-cannabis poisoning events from each year of the study (2009–2014).

Call Type	Year						
	2009	2010	2011	2012	2013	2014	Total
**Non-Cannabis**	21,119	21,452	21,615	21,746	22,621	23,226	21,119
**Cannabis**	179	226	255	219	246	362	1,487
**Percent Cannabis calls**	0.84%	1.04%	1.17%	1.00%	1.08%	1.53%	1.12%
**Total**	21,298	21,678	21,870	21,965	22,867	23,588	133,266

^a^Animal Poison Control Center.

There were slightly more female dog related calls than male related calls, with very few calls being made where the sex of the dog was unknown (Table 2). The largest group of dogs were toy and sporting breed classes (i.e., >20% of dogs) with very little representation from the FSS breed class (Table 2). The majority of calls to the APCC were made by dog owners and most calls concerned neutered dogs (Table 2). Due to the need for subsequent analyses, most variables related to community characteristics were categorized into quantiles (Table 3). In terms of state legislation, most calls came from states where cannabis possession was legally restricted at the time the call was made (Table 3). Calls reported to the APCC largely came from counties with heavily urban populations (Table 4) with an index of ethnic diversity close to 1 (Table 4).

**Table 2:** The characteristics of calls made on behalf of dogs from the US reporting poisoning events to the APCC^a^ (2009–2014).

Parameter	Frequency	Percentage of dataset
**Age (years)**	N = 132,435	
Low (< 1.1)	43,697	33.00
Medium (1.1–4.5)	44,258	33.42
High (> 4.5)	44,480	33.59
**Weight (kg)**	N = 132,445	
Low (< 7.2)	44240	33.40
Medium (7.2–21.5)	43955	33.19
High (> 21.5)	44250	33.41
**Sex**	N = 133,266	
Female	68,517	51.41
Male	64,397	48.32
Unknown	352	0.26
**Breed Class**^b^	N = 133,266	
Herding	11,330	8.50
Hound	11,805	8.86
Foundation Stock Service	425	0.32
Non-Sporting	11,011	8.26
Sporting	30,812	23.12
Terrier	14,494	10.88
Toy	32,986	24.75
Working	11,543	8.66
Other^c^	8,860	6.65
**Reproductive Status**	N = 133,148	
Intact	27,498	20.65
Neutered	101,620	76.32
Unknown	4,030	3.03
**Source of Call**	N = 133,266	
Public	95,536	71.69
Veterinarian	37,730	28.31

^a^Animal Poison Control Center.

^b^Breed classes as defined by the American Kennel Club based on the primary breed reported.

^c^Breeds in the AnTox database that are not yet categorized into American Kennel Club breed classes.

N identifies the number of dog-associated calls in the dataset with one dog randomly selected from calls involving multiple dogs.

**Table 3:** The frequency of US dogs reported to the APCC^a^ associated with each county and state-level variable (2009–2014).

Parameter	Frequency	Percentage of dataset	Number of Counties/States*
**State Legislation**	N = 133,266		
Restricted	64,433	48.35	24–34
Restricted & Medical	25,052	18.80	10–12
Legal	43,781	32.85	7–16
**Income disparity**	N = 133,266		
Low (<0.440)	44,349	33.28	556–633
Medium (0.440–0.468)	44,623	33.48	288–347
High (>0.468)	44,294	33.24	192–246
**Bachelor’s Degree or Higher (%)**	N = 133,266		
Low (<29.1)	43,935	32.97	714–786
Medium (29.1–36.8)	44,861	33.66	177–216
High (>36.8)	44,470	33.37	133–171
**High School Diploma or Higher (%)**	N = 133,266		
Low (<86.3)	44,082	33.08	399–575
Medium (86.3–89.9)	43,978	33.00	317–369
High (>89.9)	45,206	33.92	271–389
**Unemployment Rate (%)**	N = 133,266		
Low (<7.3)	44,253	33.21	295–624
Medium (7.3–9.1)	43,596	32.71	303–339
High (>9.1)	45,417	34.08	215–548
**Did Not Work (%)**	N = 133,266		
Low (<20)	43,572	32.70	241–500
Medium (20–23.6)	45,047	33.80	288–305
High (>23.6)	44,647	33.50	368–602
**Age Dependency Ratio**	N = 133,266		
Low (<55.8)	44,455	33.36	218–265
Medium (55.8–60.0)	43,082	32.33	247–268
High (>60.0)	45,729	34.31	610–659
**Median Age (Years)**	N = 133,266		
Low (<35.7)	43,338	32.52	258–315
Medium (35.7–39.0)	44,132	33.12	289–333
High (>39.0)	45,796	34.36	514–582
**Divorced (%)**	N = 133,266		
Low (<9.0)	43,935	32.97	172–235
Medium (9.0–10.8)	42,237	31.69	307–386
High (>10.8)	47,094	35.34	526–647
**Housing Cost to Income Ratio (Median %)**	N = 133,265		
Low (<21.3)	45,300	33.99	655–785
Medium (21.3–24.2)	42,742	32.07	189–254
High (>24.2)	45,223	33.93	62–140
**Married Family Households (%)**	N = 130,437		
Low (<46.4)	44,377	33.30	194–268
Medium (46.4–53.2)	44,370	33.29	417–467
High (>53.2)	44,519	33.41	411–544
**Sex Ratio (Males per 100 Females)**	N = 133,266		
Low (<93.6)	44,330	33.99	194–955
Medium (93.6–96.9)	42,159	32.32	0–362
High (>96.9)	43,948	33.69	0–362

^a^Animal Poison Control Center.

*Only cannabis legislation was measured at the state-level & ranges reflect values assigned to counties/states from 2009–2014.

N identifies the number of dog-associated calls in the dataset with one dog randomly selected from calls involving multiple dogs.

**Table 4:** Descriptive statistics depicting county-level continuous variables (2009–2014).

Parameter	Mean	Median	Standard Deviation	Interquartile Range	N^‡^
**County Urban Population (%)**	89.07	95.83	16.78	87.40–98.79	133,266
**County Ethnic Diversity**	0.97	1.03	0.30	0.78–1.22	133,266

^‡^N identifies the number of dog-associated calls in the dataset with one dog randomly selected from calls involving multiple dogs.

### Univariable mixed logistic regression

Based on our univariable mixed logistic regression models, the following variables were significantly associated with the odds of a call being related to cannabis: i) state-level legislation, ii) county-level variables: percent urban population, ethnic diversity, income disparity, percent did not work, percent unemployment, age dependency ratio, and percent married family households, and iii) dog-level variables: weight, sex, reproductive status, breed class, call source, year the call was made (Tables 5 and 6).

**Table 5:** Results of univariable mixed logistic regression models^†^ examining associations between each individual dog-level variable on the odds of a US dog-associated poisoning call to the APCC^a^ being related to cannabis (2009–2014).

Parameter	Odds Ratio	Coefficient 95% CI	P-Value
**Year**	1.10	1.06; 1.13	<0.001
**Weight (kg)**			
Low (<7.2)	Referent		
Medium (7.2–21.5)	0.75	0.67; 0.85	<0.001
High (>21.5)	0.64	0.56; 0.73	<0.001
**Sex**			
Female	Referent		
Male	1.21	1.09; 1.34	<0.001
Unknown	1.13	0.42; 3.04	0.807
**Reproductive Status**			
Intact	Referent		
Neutered	0.78	0.69; 0.88	<0.001
Unknown	0.90	0.67; 1.22	0.497
**Breed Class**			
Herding	Referent		
Hound	1.08	0.84; 1.38	0.564
Foundation Stock Service	0.23	0.032; 1.63	0.141
Non-Sporting	1.20	0.94; 1.53	0.145
Sporting	0.70	0.56; 0.88	0.002
Terrier	1.24	0.99; 1.57	0.063
Toy	1.30	1.06; 1.59	0.012
Working	0.86	0.66; 1.12	0.261
Other	1.09	0.83; 1.42	0.543
**Source of Call**			
Public	Referent		
Veterinarian	1.61	1.45; 1.78	<0.001
**Age (years)**			
Low (< 1.1)	Referent		
Medium (1.1–4.5)	0.95	0.84; 1.08	0.434
High (> 4.5)	1.01	0.89; 1.15	0.826

^a^Animal Poison Control Center.

^†^Models include random intercepts for county and state.

**Table 6:** Results of univariable mixed logistic regression models^†^ examining the associations between each individual community-level variable on the odds of a US dog-associated poisoning call to the APCC^a^ being related to cannabis (2009–2014).

Parameter	Odds Ratio	95% Confidence Intervals	P-Value
**State Legislation**			
Restricted	Referent		
Restricted & Medical	1.39	1.14; 1.70	0.001
Legal	1.77	1.47; 2.14	<0.001
**County Urban Population (%)**	1.01	1.004; 1.01	<0.001
**County Ethnic Diversity**	1.84	1.45; 2.33	<0.001
**Income disparity**			
Low (<0.440)	Referent		
Medium (0.440–0.468)	1.17	1.01; 1.36	0.042
High (>0.468)	1.46	1.25; 1.71	<0.001
**Bachelor’s Degree or Higher (%)**			
Low (<29.1)	Referent		
Medium (29.1–36.8)	1.12	0.96; 1.30	0.142
High (>36.8)	1.13	0.96; 1.32	0.131
**High School Diploma or Higher (%)**			
Low (<86.3)	Referent		
Medium (86.3–89.9)	0.95	0.82; 1.11	0.538
High (>89.9)	0.92	0.78; 1.09	0.342
**Unemployment Rate (%)**			
Low (<7.3)	Referent		
Medium (7.3–9.1)	1.16	1.003; 1.33	0.045
High (>9.1)	1.26	1.08; 1.47	0.004
**Did Not Work (%)**			
Low (<20)	Referent		
Medium (20–23.6)	1.26	1.08; 1.48	0.004
High (>23.6)	1.43	1.20; 1.69	<0.001
**Age Dependency Ratio**			
Low (<55.8)	Referent		
Medium (55.8–60.0)	0.89	0.76; 1.03	0.112
High (>60.0)	0.85	0.73; 0.99	0.039
**Median Age (Years)**			
Low (<35.7)	Referent		
Medium (35.7–39.0)	0.90	0.77; 1.06	0.201
High (>39.0)	0.94	0.79; 1.12	0.506
**Divorced (%)**			
Low (<9.0)	Referent		
Medium (9.0–10.8)	0.88	0.75; 1.04	0.130
High (>10.8)	0.92	0.76; 1.11	0.381
**Housing Cost to Income Ratio (Median %)**			
Low (<21.3)	Referent		
Medium (21.3–24.2)	1.10	0.94; 1.30	0.226
High (>24.2)	1.19	0.99; 1.45	0.070
**Married Family Households (%)**			
Low (<46.4)	Referent		
Medium (46.4–53.2)	0.85	0.73; 0.99	0.031
High (>53.2)	0.78	0.67; 0.90	0.001
**Sex Ratio (Males per 100 Females)**			
Low (<93.6)	Referent		
Medium (93.6–96.9)	1.02	0.88; 1.19	0.805
High (>96.9)	1.05	0.90; 1.24	0.873

^a^Animal Poison Control Center.

^†^Models include random intercepts for county and state.

### Multivariable mixed logistic regression

The following variables were included in our multivariable mixed logistic regression model: state-level legislation, county-level income disparity and percent urban population, as well as dog-level weight, sex, reproductive status, breed class, call source, and year the call was made (Table 7).

**Table 7:** Results of multivariable mixed logistic regression model^†^ examining the associations between each dog-level and community-level variable on the odds of a poisoning call to the APCC^a^ being related to cannabis (2009–2014).

Parameter	Odds Ratio	95% Confidence Intervals	P-Value
**State Legislation**			
Restricted	Referent		
Restricted & Medical	1.36	1.11; 1.68	0.004
Legal	1.59	1.28; 1.97	<0.001
**Year**	1.07	1.04; 1.11	<0.001
**Income disparity**			
Low (<0.440)	Referent		
Medium (0.440–0.468)	1.07	0.91; 1.25	0.407
High (>0.468)^b^	1.26	1.07; 1.48	0.005
**Source of Call**			
Public	Referent		
Veterinarian	1.62	1.45; 1.810	<0.001
**County Urban Population (%)**	1.006	1.001; 1.01	0.011
**Weight (kg)**			
Low (<7.2)	Referent		
Medium (7.2–21.5)	0.82	0.71; 0.95	0.008
High (>21.5)	0.82	0.69; 0.99	0.036
**Breed Class**			
Herding	Referent		
Hound	1.01	0.78; 1.31	0.934
Foundation Stock Service	0.22	0.03; 1.59	0.133
Non-Sporting	1.10	0.85; 1.41	0.484
Sporting	0.71	0.57; 0.89	0.003
Terrier	1.17	0.92; 1.48	0.192
Toy	1.07	0.85; 1.36	0.549
Working	0.85	0.65; 1.11	0.222
Other	0.998	0.76; 1.31	0.989
**Reproductive Status**			
Intact	Referent		
Neutered	0.84	0.74; 0.95	0.007
Unknown	0.90	0.66; 1.23	0.509
**Sex**			
Female	Referent		
Male	1.20	1.08; 1.33	0.001
Unknown	0.78	0.19; 3.25	0.734
**Random Effects**	**Variance**	**95% Confidence Intervals**	
State	0.041	0.014; 0.13	
County	0.024	0.005; 0.12	

^a^Animal Poison Control Center.

^b^Odds of a cannabis call is significantly greater for high income disparity vs medium (OR = 1.18; 95%CI = 1.02–1.36; p-value = 0.023).

^†^Model includes random intercept for county and state.

#### I) State-level cannabis legislation

The odds of a call being related to a cannabis poisoning event was greater for dogs living in states where cannabis possession was legal or restricted but medically permitted compared to dogs in states where cannabis possession was restricted (Table 7).

#### II) County-level variables

There were higher odds of a cannabis poisoning call in counties with high income disparity compared to medium and low income disparity counties (Table 7). There was also a positive linear association between the percent urban population of a county and the odds of a call to the APCC being associated with a cannabis poisoning (Table 7).

#### III) Dog-level variables

There was a statistically significant increase in the odds of a cannabis poisoning call to the APCC over the years of the study (Table 7). In addition, the odds of a call concerning a cannabis poisoning event were significantly greater if the call came from a veterinarian rather than a dog’s owner (Table 7).

The odds of a call being related to a dog cannabis poisoning event were significantly greater for male dogs than female dogs. Similarly, the odds of a dog cannabis call were significantly greater for intact dogs than neutered dogs (Table 7). There were lower odds of a cannabis call for large and medium sized dogs compared to small sized dogs (Table 7). The odds of a cannabis call were significantly lower for sporting breeds compared to herding, hound, non-sporting, terrier, toy, and other breeds. Similarly, the odds of a cannabis call were significantly lower for working breeds than terrier breeds (Table 8).

**Table 8:** Table of contrasts* examining the associations between each breed class on the odds of a poisoning call to the APCC^a^ being related to cannabis (2009–2014).

Breed Class	Herding	Hound	Foundation Stock Service	Non-Sporting	Sporting	Terrier	Toy	Working	Other
Herding	-	-	-	-	-	-	-	-	-
Hound	OR: 1.01	-	-	-	-	-	-	-	-
	95%CI: 0.78; 1.31								
	P-value: 0.934								
Foundation Stock Service	OR: 0.22	OR: 0.22	-	-	-	-	-	-	-
	95%CI: 0.03; 1.59	95%CI: 0.03; 1.57							
	P-value: 0.133	P-value: 0.130							
Non-Sporting	OR: 1.10	OR: 1.08	OR: 4.96	-	-	-	-	-	-
	95%CI: 0.85; 1.41	95%CI: 0.85; 1.38	95%CI: 0.69; 35.61						
	P-value: 0.484	P-value: 0.515	P-value: 0.111						
Sporting	**OR: 0.71**	**OR: 0.70**	OR: 3.22	**OR: 0.65**	-	-	-	-	-
	**95%CI: 0.57; 0.89**	**95%CI: 0.56; 0.89**	95%CI: 0.45; 23.02	**95%CI: 0.52; 0.82**					
	**P-value: 0.003**	**P-value: 0.003**	P-value: 0.244	**P-value: <0.001**					
Terrier	OR: 1.17	OR: 1.16	OR: 5.30	OR: 1.07	**OR: 1.65**	-	-	-	-
	95%CI: 0.92; 1.48	95%CI: 0.92; 1.45	95%CI: 0.74; 37.95	95%CI: 0.86; 1.33	**95%CI: 1.34; 2.02**				
	P-value: 0.192	P-value: 0.208	P-value: 0.097	P-value: 0.559	**P-value: <0.001**				
Toy	OR: 1.07	OR: 1.06	OR: 4.87	OR: 0.98	**OR: 1.51**	OR: 0.92	-	-	-
	95%CI: 0.85; 1.36	95%CI: 0.86; 1.31	95%CI: 0.68; 34.82	95%CI: 0.81; 1.19	**95%CI: 1.23; 1.86**	95%CI: 0.76; 1.11			
	P-value: 0.549	P-value: 0.568	P-value: 0.115	P-value: 0.846	**P-value: <0.001**	P-value: 0.369			
Working	OR: 0.85	OR: 0.84	OR: 3.83	OR: 0.77	OR: 1.19	**OR: 0.72**	OR: 0.79	-	-
	95%CI: 0.65; 1.11	95%CI: 0.63; 1.10	95%CI: 0.53; 27.57	95%CI: 0.59; 1.02	95%CI: 0.94; 1.51	**95%CI: 0.56; 0.93**	95%CI: 0.61; 1.02		
	P-value: 0.222	P-value: 0.209	P-value: 0.182	P-value: 0.066	P-value: 0.146	**P-value: 0.013**	P-value: 0.069		
Other	OR: 0.998	OR: 0.99	OR: 4.52	OR: 0.91	**OR: 1.41**	OR: 0.85	OR: 0.93	OR: 1.18	-
	95%CI: 0.76; 1.31	95%CI: 0.76; 1.29	95%CI: 0.63; 32.53	95%CI: 0.70; 1.18	**95%CI: 1.10; 1.79**	95%CI: 0.67; 1.09	95%CI: 0.74; 1.17	95%CI: 0.89; 1.57	
	P-value: 0.989	P-value: 0.925	P-value: 0.134	P-value: 0.485	**P-value: 0.006**	P-value: 0.205	P-value: 0.539	P-value: 0.257	

^a^Animal Poison Control Center.

Referent breed class used in the column heading.

Bolded cells indicate the relationship between the specific breed class and the referent has a p-value < 0.05.

*Contrasts based on model presented in Table 7.

The variance partition coefficients indicate that 98.06%, 0.72%, and 1.23% of the variance was explained at the dog, county, and state-levels, respectively (Table 7). The best linear unbiased predictions (BLUPs) met homoscedasticity and normality assumptions, and no outliers were identified.

## Discussion

This study provides a national (US) population-based analysis aimed at identifying dog and community-level factors associated with cannabis poisonings in dogs. The dog-level portion of our dataset consisted of call information provided by the ASPCA concerning dog cannabis poisonings, which were collected at the time of enquiry with much of the information detailed by the caller (owner or veterinarian). This information was combined with county-level socioeconomic data, as well as state-level cannabis legislation data to also study the effects of community-level predictor variables on cannabis poisoning in dogs. A mixed logistic regression model with random intercepts for county and state was fit to the data and identified several dog and community-level factors that were associated with the odds of a call being related to dog cannabis poisoning events.

### Community-level variables

Analyses showed that as state-level penalties for cannabis possession decreased, the odds of a cannabis poisoning call to the APCC increased. This association may infer that as cannabis legislation relaxes, the frequency of dog cannabis poisoning increases. A similar relationship was previously reported between eased cannabis legislation and increased cannabis exposures in children [20]. The relationship between relaxed legislation and increased dog cannabis poisoning calls may also be related to increased cannabis use or changes in the types of cannabis products used, such as edibles (i.e., foods containing added cannabinoids). Edible cannabis is of particular concern as it is more likely to be accidentally ingested and/or over-consumed by humans, due to poor understanding of the delayed onset of the psychoactive effects of cannabis ingestion [32–34]. However, it is also possible that the association between relaxed legislation and increased dog cannabis poisonings reflects a reduction of fear from the owners to report a dog poisoning from a substance that was previously associated with illegal drug possession.

The odds of a cannabis poisoning call to the APCC increased as income disparity of the county increased. This relationship may reflect that as income disparity in the county increases, more dogs are exposed to cannabis. There is some evidence to support that cannabis use is higher in humans where there is higher income disparity [35], and its use is also associated with lower income and financial instability [23, 26].

There was a positive linear association between the percent urban population of a county and the odds of a cannabis related call to the APCC. This finding supports other studies that have also reported that the use and abuse of cannabis in humans is higher in urban centers compared to rural [36, 37]. Consequently, our results could reflect that since there is more cannabis and cannabis use in urban environments, dogs in urban areas are at a greater risk of being exposed to cannabis products. When considering the effect of the percent urban population variable on the odds of a call being related to cannabis, it is important to note that a variable like income disparity could act as an intervening variable hence the full effect of the percent urban population variable may not be captured in the final multivariable model (Fig 1).

### Dog-level variables

The odds of a call to the APCC due to cannabis poisoning increased throughout the study, a trend also identified in a previous study [3]. This may suggest an increase in dog cannabis poisonings throughout the period of this study, possibly due to the increasing popularity/accessibility of cannabis, especially of edible products [38, 39]. This change in calling patterns may also reflect the continual increase in THC concentration in cannabis products [40]. This temporal trend contrasts with our earlier work with APCC data where opioid poisonings in dogs decreased during the same period [28].

There were higher odds of a cannabis call coming from a veterinarian compared to a call coming from a dog owner. Similar to the legislation variable and our previous work with dog opioid poisonings [28], this could reflect a lack of willingness of owners to report cannabis poisoning events due to the stigma associated with cannabis use.

Male and intact dogs are at higher odds than female and neutered dogs of being involved in a cannabis poisoning call to the APCC. This is similar to our findings concerning calls related to opioid poisoning [28]. This may be due to behavioural differences between male and female dogs and intact and neutered dogs [41, 42]. The breed class variables suggest that sporting breed class dogs had a lower odds of being involved in a cannabis poisoning call than hound, non-sporting, terrier, toy, and other breeds, while working breed dogs had a lower odds than terrier breeds. These relationships could be reflecting behavioural differences between the different breed classes that put them at a higher risk of a severe cannabis poisoning event. It is also possible that the attitudes of owners concerning neutering and the decision to select a specific breed or sex of dog are associated with cannabis use or calling behaviour.

It appears that size also influences the odds of a call being related to cannabis. Small dogs have higher odds of being involved in a cannabis related call than medium and large dogs. This relationship may reflect the way smaller dogs are handled, giving them better access to cannabis, causing them to be exposed more often. It could also reflect that smaller dogs need a lower dose of cannabis to have a negative response that warrants a call to the APCC. Owners could also perceive smaller dogs as more vulnerable to cannabis, prompting owners to call the APCC once it is known that cannabis has been consumed or clinical signs are presented. A similar relationship was found with our work concerning opioids [28].

Potential systematic biases must be considered when interpreting our results. As the services to the APCC cost 65 USD per case, non-response bias could occur if the associations measured among people using the service are different from those who do not or cannot afford to use the service. However, including socioeconomic variables in our model corrects some of the inherent non-response biases present in our data as a result of the service cost. Furthermore, since much of the information was provided to the APCC by the caller, and toxicants were not confirmed through laboratory testing, some misclassification of the toxicant may be present in the dataset.

The associations at the dog-level such as weight, reproductive status, and call source show there are several similarities in the exposure dynamics of cannabis and opioids. Extra caution could be used to protect dogs with certain characteristics from exposure to cannabis and opioids. It also appears that while opioid poisoning events in dogs are declining, poisoning events involving cannabis products are increasing. This may reflect changing attitudes and usage patterns of these drugs/products in human populations. Therefore, strategies aimed at remedying this situation will need a broad One Health perspective, targeted towards protecting the dogs at highest risk, but also looking beyond animal-level characteristics and managing the use and misuse of these substances by humans.

## Conclusion

As human cannabis use continues to increase and is accepted as a societal norm, it is important to understand how these changes will impact all populations. By identifying dog and community-level characteristics that impact cannabis calls to the APCC, this study adds to the growing One Health discourse concerning the connected health of humans and animals. By including the impact of human drug policy on dog health this study could provide information to other governing bodies, public health departments, and veterinarians to prepare other regions of the world for changes in cannabis legislation following similar trends to those seen in the US. As state penalty is decreased or removed for cannabis possession, we found the odds of a cannabis poisoning call increased. This information may help poison control centers and veterinarians responding to cannabis poisonings prepare for future legislative changes that will likely occur in other countries and US states. This study highlights that education about safeguarding recreational cannabis intended for human consumption needs to be considered to protect vulnerable populations. Additionally, it indicates that the protection of vulnerable populations should be considered during changes to legislation. In our study cannabis calls increased in proportion and in total numbers relative to all other calls. There does appear to be a general increasing trend in severe cannabis poisoning events in pet dogs in the US. This trend highlights the growing need to understand the effects of human cannabis use on pet dogs and a need to educate the public and veterinary communities on identifying and providing care for cannabis poisonings. Based on our study, we conclude that the incidence of these cannabis poisoning events is affected by animal and socioeconomic characteristics as well as legislation. This study is intended to play a role in increasing awareness among the public and veterinary communities of the effects of recreational drug use on dog populations, and highlights the need to educate dog owners before legislation changes to decriminalize or legalize cannabis products are enacted.
